# Supplementary material for: Specific c-Jun N-Terminal Kinase Inhibitor, JNK-IN-8 Suppresses Mesenchymal Profile of PTX-Resistant MCF-7 Cells through Modulating PI3K/Akt, MAPK and Wnt Signaling Pathways
Source: Biology (Basel). 2020 Oct 1;9(10):320. doi: 10.3390/biology9100320 (PMC7599514; doi:10.3390/biology9100320)
Supplement: Supplementary file 1 [file biology-09-00320-s001.pdf]

biology

Supplementary material

Specific c-Jun N-Terminal Kinase Inhibitor, JNK-IN-8 Suppresses Mesenchymal Profile of PTX-Resistant MCF-7 Cells through Modulating PI3K/Akt, MAPK and Wnt Signaling Pathways

Pelin Ozfiliz-Kilbas, Ozlem Sonmez, Pinar Uysal-Onganer, Ajda Coker-Gurkan, Pinar Obakan-Yerlikaya and Elif Damla Arisan

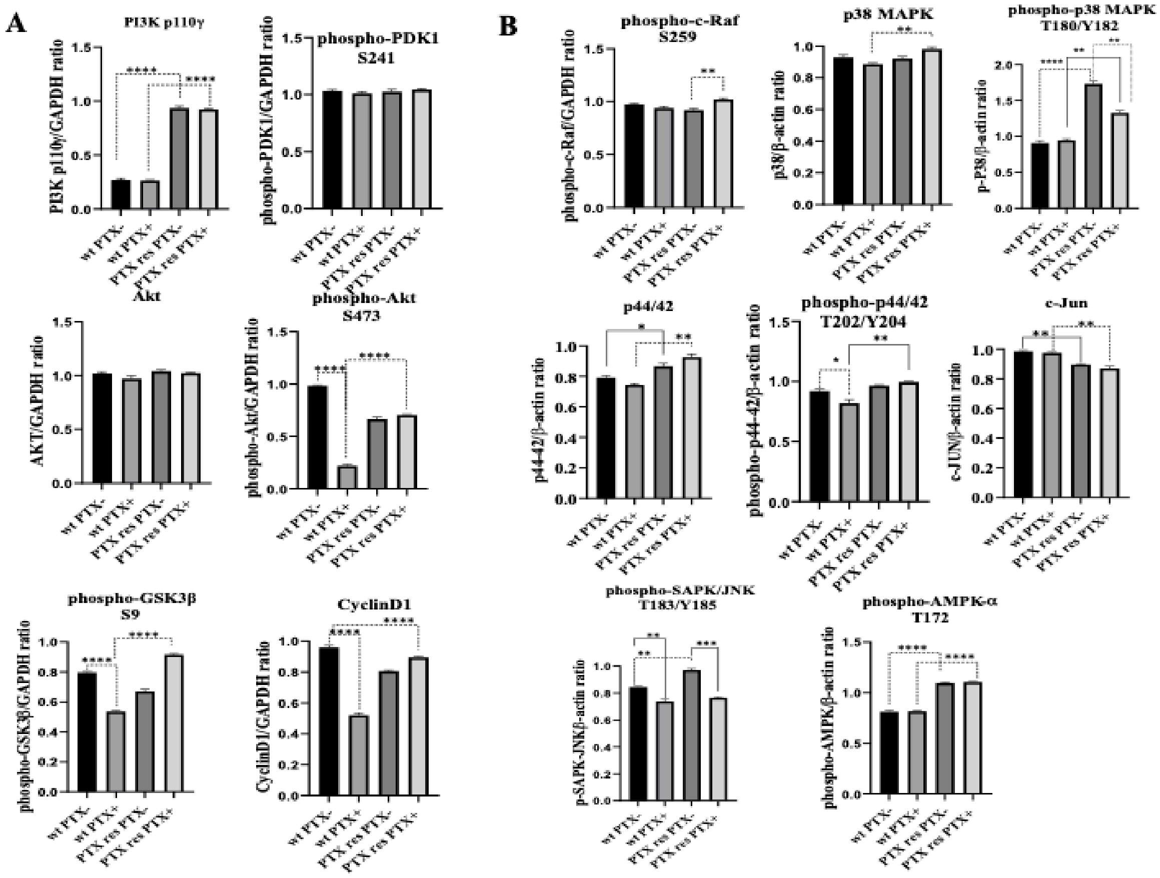

Figure S1. Densitometry analysis of PI3K/Akt (**A**) and MAPK (**B**) pathway members demonstrated in Figure 3. Statistically significant values considered as following: A) PI3K, phospho-Akt S473, phospho- GSK3β S9 and Cyclin D1 **** $p < 0.0001$. B)phospho-c-RAF ** $p = 0.0049$, p38 ** $p = 0.0060$, phospho-p38 ** $p = 0.0013$, p44/42 * p= 0.0381, phospho-p44/42 * = 0.0130, ** $p = 0.0016$, c-Jun ** $p = 0.0023$ phospho-SAPK/JNK ** $p = 0.0013$, phospho-AMPK-α T172 **** $p < 0.0001$.

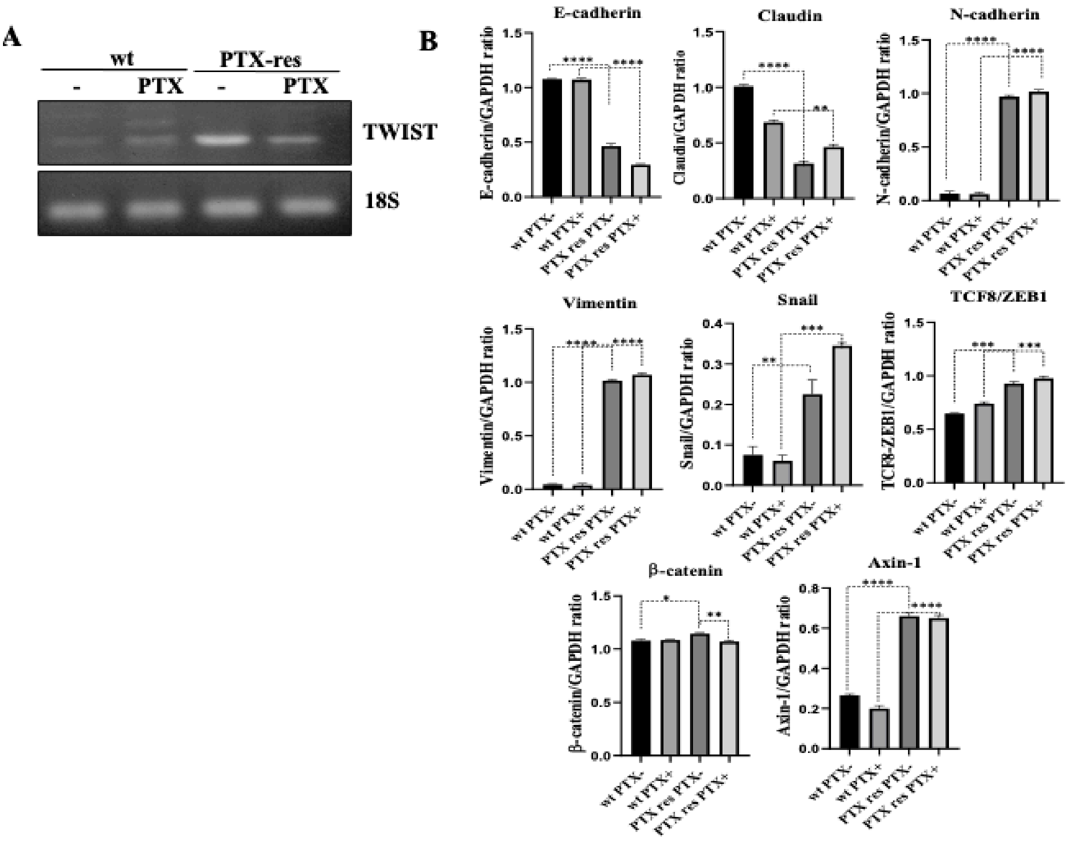

Figure S2. (**A**) The mRNA expression levels of Twist in wt and PTX-res cells determined by RT-PCR. 18S was used as a loading control. (**B**) Densitometry analysis of EMT pathway members demonstrated in Figure 4. Statistically significant values considered as following: E-cadherin **** $p < 0.0001$, Claudin ** $p = 0.0013$, **** $p < 0.0001$, N-cadherin and Vimentin **** $p < 0.0001$, Snail ** $p = 0.0084$, *** $p = 0.0007$, TCF8/ZEB1 *** $p = 0.0003$, β-catenin * $p = 0.0088$, Axin-1**** $p < 0.0001$.

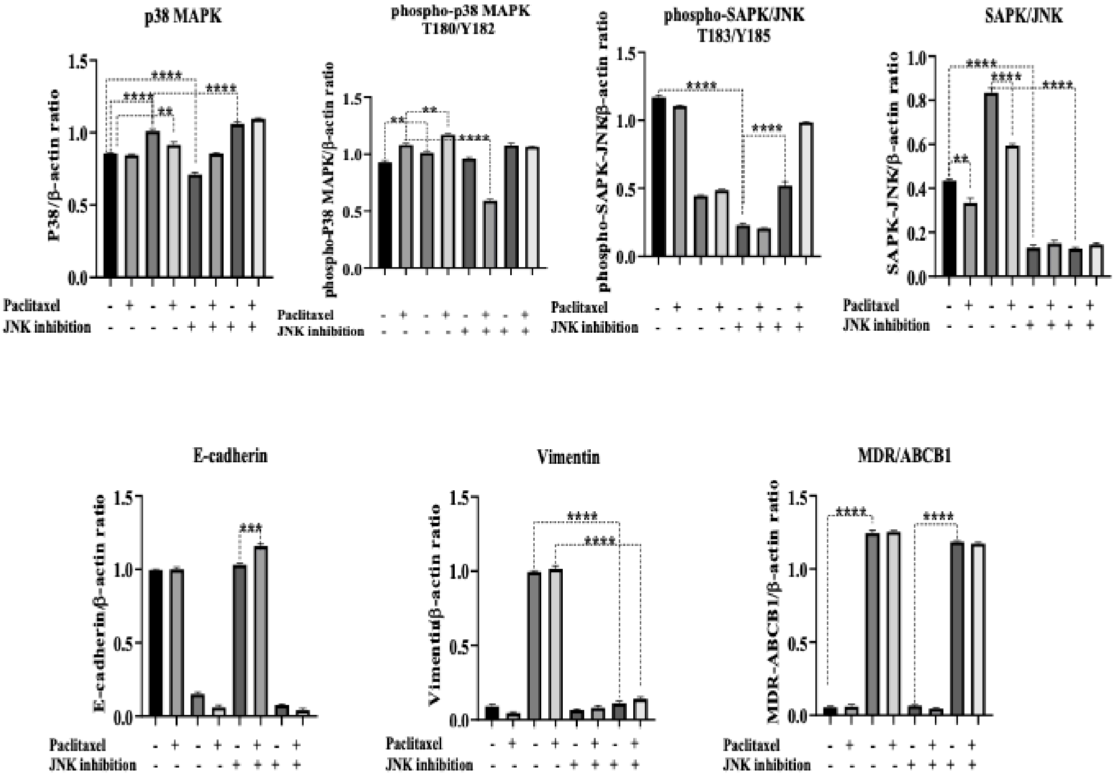

Figure S3. Densitometry analysis of p38, phospho-p38, SAPK/JNK, phospho-SAPK/JNK, E-cadherin, Vimentin, and MDR/ABCB1 expressions demonstrated in Figure 5. Statistically significant values considered as following: p38 **** $p < 0.0001$, ** $p = 0.0036$, phospho-p38-MAPK Thr180/Tyr182 ** $p = 0.0079$, **** $p < 0.0001$, phospho-SAPK/JNK, SAPK/JNK **** $p < 0.0001$, E-cadherin *** $p = 0.0001$, Vimentin and MDR/ABCB1 **** $p < 0.0001$.

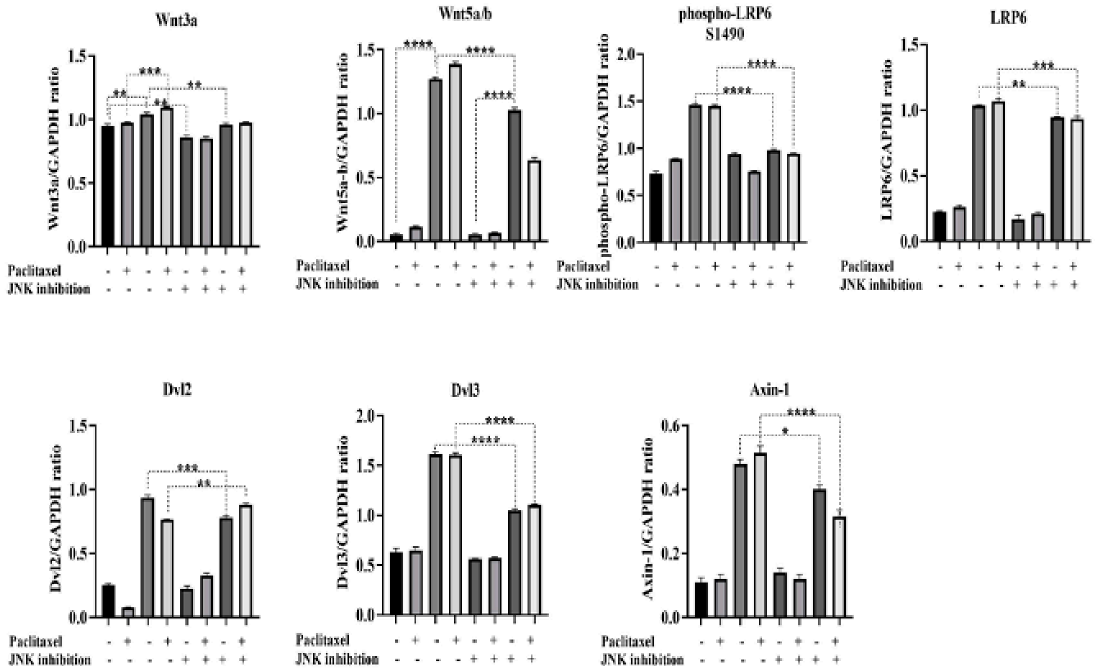

Figure S4. Densitometry analysis of Wnt signaling members demonstrated in Figure 6. Statistically significant values considered as following: Wnt3a ** $p = 0.0010$, *** $p = 0.0001$, Wn5a/b, phospho- LRP6 Ser1490 **** $p < 0.0001$, LRP6 ** $p = 0.0063$, *** $p = 0.0004$, Dvl2 ** $p = 0.0014$, *** $p = 0.0002$, Dvl3 **** $p < 0.0001$, Axin1 **** $p < 0.0001$, * $p = 0.0150$.

© 2020 by the authors. Licensee MDPI, Basel, Switzerland. This article is an open access article distributed under the terms and conditions of the Creative Commons Attribution (CC BY) license (http://creativecommons.org/licenses/by/4.0/).
